# Non-Coding RNAs in Castration-Resistant Prostate Cancer: Regulation of Androgen Receptor Signaling and Cancer Metabolism

**DOI:** 10.3390/ijms161226138

**Published:** 2015-12-04

**Authors:** Jing-Wen Shih, Ling-Yu Wang, Chiu-Lien Hung, Hsing-Jien Kung, Chia-Ling Hsieh

**Affiliations:** 1 Integrated Translational Lab, The Center of Translational Medicine, Taipei Medical University, Taipei 11031, Taiwan; shihjw@tmu.edu.tw (J.-W.S.); hkung@nhri.org.tw (H.-J.K.); 2 The Ph.D. Program for Translational Medicine, College of Medical Science and Technology, Taipei Medical University, Taipei 11031, Taiwan; 3 Department of Biochemistry and Molecular Medicine, Comprehensive Cancer Center, University of California at Davis, Sacramento, CA 95817, USA; lywang@ucdavis.edu (L.-Y.W.); clhung@ucdavis.edu (C.-L.H.); 4 Institute of Molecular and Genomic Medicine, National Health Research Institutes, Zhunan, Miaoli County 35053, Taiwan

**Keywords:** non-coding RNA, micro RNAs, long non-coding RNAs, castration-resistant prostate cancer, androgen receptor, cancer metabolism

## Abstract

Hormone-refractory prostate cancer frequently relapses from therapy and inevitably progresses to a bone-metastatic status with no cure. Understanding of the molecular mechanisms conferring resistance to androgen deprivation therapy has the potential to lead to the discovery of novel therapeutic targets for type of prostate cancer with poor prognosis. Progression to castration-resistant prostate cancer (CRPC) is characterized by aberrant androgen receptor (AR) expression and persistent AR signaling activity. Alterations in metabolic activity regulated by oncogenic pathways, such as c-Myc, were found to promote prostate cancer growth during the development of CRPC. Non-coding RNAs represent a diverse family of regulatory transcripts that drive tumorigenesis of prostate cancer and various other cancers by their hyperactivity or diminished function. A number of studies have examined differentially expressed non-coding RNAs in each stage of prostate cancer. Herein, we highlight the emerging impacts of microRNAs and long non-coding RNAs linked to reactivation of the AR signaling axis and reprogramming of the cellular metabolism in prostate cancer. The translational implications of non-coding RNA research for developing new biomarkers and therapeutic strategies for CRPC are also discussed.

## 1. Introduction

Prostate cancer (PC) is the most commonly diagnosed malignancy among men in Western countries, continually ranking as the second leading cause of cancer mortality in that group. It was estimated that there were 220,800 newly diagnosed cases and 27,540 deaths in the USA attributable to PC in 2015 [1]. Advances in detection techniques, such as magnetic resonance imaging [2,3] and prostate-specific antigen (PSA) screening [4,5] have led to PC diagnoses at earlier stages, at which patients are typically treated with surgery, radiation, and in some cases, active surveillance only, and a majority of them are successfully cured of the disease [6,7,8]. The development and progression of PC depend on androgenic stimulation [9]. As such, for patients who present with locally advanced or disseminated PC, and biochemical recurrence after treatment for localized PC, androgen-deprivation therapy (ADT) is considered a key treatment as monotherapy or in combination with other regimens. Most patients initially respond to ADT; however, the intrinsic nature of the heterogeneity of tumor cells results in resistance to treatment and progression into highly morbid disease termed castration-resistant prostate cancer (CRPC). Significantly, bone metastases often occur in 90% of men with CRPC and can produce serious morbidity with the development of severe pain, pathological fractures and spinal cord compression, cranial neuropathies related to bone metastasis from syndromes at the base of the skull, and anemia [10,11]. Among the available therapeutic approaches for treating CRPC, conventional chemotherapy causes unpleasant side effects and offers a survival time of less than 19 months [12,13,14]. Several agents that target distinct mechanisms of action, including tubulin targeting chemotherapy (cabazitaxel), immunotherapy (sipuleucel-T), the androgen biosynthesis inhibitor (abiraterone), androgen receptor (AR) antagonist (enzalutamide), and α-emitting radiotherapy (radium-223), have recently been introduced into clinics and have shown promising results [15]. Despite these advancements, metastatic CRPC (mCRPC) remains an incurable disease [16]. Therefore, delineating the molecular mechanisms that contribute to PC cell growth in androgen-depleted conditions will provide the basis for developing future CRPC therapies.

Emerging evidence revealed that in human, <2% of the total genome sequence encodes transcripts which are protein-coding; the rest of it is also actively transcribed, generating a vast quantity of functional non-coding RNAs (ncRNAs) [17,18,19], which are alternatively spliced and/or processed into smaller products with little or no protein-coding capacity [20,21]. These ncRNAs are typically classified into small and long ncRNAs (lncRNAs) based on the size range of <200 or >200 nucleotides, respectively [22], and interact with DNA, RNA, and protein molecules to engage in diverse regulatory activities, including chromatin remodeling [23], RNA splicing and editing [24], translational inhibition [25], and messenger (m)RNA destruction [26]. Among the different classes of non-coding transcripts, microRNAs (miRNAs) bind to the 3′-untranslated region (3′-UTR) of their target genes, which include key transcription factors, receptors, and kinases, to suppress protein translation via non-perfect pairing of just six to eight nucleotides in length [27] or induce mRNA degradation in the case of perfect complementarity with the target site of approximately 22 nucleotides [28]. After their initial identification [29], miRNAs were found to play integral roles in regulating an array of fundamental cellular processes, including the cell cycle, differentiation, and proliferation [30], thereby tuning numerous pathways related to development and diseases [31].

Unlike small ncRNAs, lncRNAs are poorly conserved among different species and are usually expressed at low basal levels, making them look like transcriptional noise [32,33]. However, additional genetic studies estimated the number of human lncRNAs to be in the range of 7000–23,000 [34], and some of them have emerged as important gene regulators at several levels of cellular processes in a range of physiological and pathological situations, including cancer. Like miRNA, the major function of lncRNAs thus far studied is involvement in the transcriptional or posttranscriptional regulation of gene expression [35]. At the transcription level, lncRNAs can act as molecular chaperons or scaffolds for the delivery of transcriptional factors to the chromatin site, the recruitment of histone-modifying complexes to regulate chromatin conformation, or the connection with distal gene-regulatory elements to effectively modulate transcription of targeted loci [35]. Alterations of cancer-related lncRNAs including aberrant expression and single-nucleotide polymorphisms (SNPs) were shown to facilitate tumor-cell proliferation, invasion, and survival during cancer development and progression [34,36,37]. Despite increasing numbers of cancer-related lncRNAs having been identified, their biological mechanisms underlying cancer development remain largely unknown.

Although the CRPC is unresponsive to androgen deprivation, the AR signaling axis is persistently activated and plays a pivotal role in the growth and survival of hormone-refractory PC cells. Accordingly, both AR itself and the signaling processes downstream of the receptor remain attractive targets for treating CRPC. Efforts to identify the key downstream targets of the AR in PC have found links to metabolic pathways [38,39,40]. Further functional studies have also provided evidence for a connection between the AR signaling and the reprogramming of cellular metabolism [41,42,43], an emerging hallmark of cancer cell growth and survival under adverse conditions [44]. It is particularly interesting that in CRPC where aberrant AR activation is often observed, the cancer cell can be still fueled by anabolic biosynthesis [40]. Based on an early study reporting suppressed glucose metabolism in PC patients upon ADT [45], it is possible that alterations in cellular metabolism may contribute to the development of CRPC and metastasis. This speculation is supported by recent studies showing that nuclear factor (NF)-κB-mediated upregulation of glucose metabolism promotes CRPC cell growth [46]; while sustained carbon flux to the pentose phosphate pathway [42] and leucine addiction with elevated expression of amino acids transporters [47] exhibit the capacity to mediate cancer cell growth during the progression to castration resistance and drive metastasis. Given that AR induces a distinct transcriptional program in CRPC [48], identifying differentially expressed ncRNAs and their targets that cooperate with AR signaling and metabolic pathways to the corresponding response during the progression of CRPC offers new opportunities to obtain molecular signatures for classification, early diagnosis, and discover potential therapeutic targets of this disease.

A number of studies have examined differentially expressed ncRNAs in each stage of PC using established cell lines and patient samples taken from prostate tissues, blood, serum, or urine. Herein, we highlight the emerging impacts of miRNAs and lncRNAs linked to reactivation of the AR signaling axis and reprogramming of the cellular metabolism which are known critical mechanisms implicated in the development of CRPC. Translational implications of ncRNA research for developing new biomarkers and therapeutic strategies for CRPC are also discussed.

## 2. miRNAs in the Androgen Receptor (AR) Signaling Pathway

**Figure 1:**
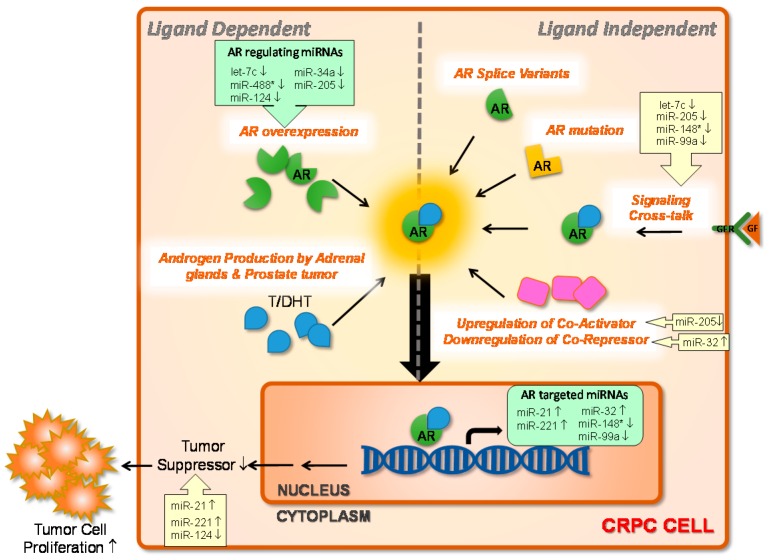
Androgen receptor (AR) signaling and mechanisms of castration resistance. A number of microRNAs (miRNAs) that are either regulated by the AR (AR-targeted miRNAs) or directly bound to the AR mRNA 3′-untranslated region (AR-regulating miRNAs) invloved in the reactivation of AR signaling axis in castration-resistant prostate cancer (CRPC) are indicated and discussed in the text of the present article. T, testosterone; DHT, dihydrotestosterone; GF, growth factor; GFR, growth factor receptor; ↑, upregulation; ↓, downregulation.

Empirical studies have illustrated six potential mechanisms of continued AR signaling in CRPC (Figure 1): (1) amplification of the AR, leading to higher sensitivity of the AR to its agonists [49,50]; (2) somatic mutations of the AR that allow the use of a wider spectrum of ligands [51,52,53]; (3) upregulation of intratumoral androgen biosynthesis, leading to a shift from paracrine stromal growth support to an autocrine mode [54,55,56]; (4) the emergence of altered splice variants of the AR which are devoid of a ligand-binding domain and become constitutively active [57,58,59,60,61]; (5) altered transcriptional activity of the AR due to changes in the balance of AR coregulators [62,63] or post-translational alterations of AR, especially phosphorylation that stabilizes the AR and protects it from proteolytic degradation [64,65,66,67]; and (6) cross-talk with other oncogenic signaling pathways via activation or inhibition of downstream signaling molecules or target genes common to both pathways, allowing ligand-independent AR action [68,69,70,71]. Through reactivation of the AR signaling pathway during hormone therapy, the balance between the proliferation and apoptotic cell death of PC cells is disturbed, which further drives tumor progression toward castration resistance. The AR is a DNA-binding transcription factor which regulates genes and ncRNAs. At the same time, the AR has a long, relatively AU-rich 3′-UTR, which is predicted to be a target of many miRNAs. Differential expressions of miRNAs in CRPC cells and tissues are being intensively investigated and extensively reviewed [72,73,74,75,76]. Dysregulated miRNAs that are involved in AR regulation and reactivation during CRPC progression (Figure 1) are individually discussed below.

### 2.1. Androgen-Regulated miRNAs

The AR activates or represses the transcription of androgen-responsive genes via binding to androgen-responsive elements that are usually located in the 5′ flanking region of its target genes. By integrating expression profile data from clinical tissues with chromatin immunoprecipitation sequencing (ChIP-seq) data of AR-binding sites (ARBSs) in androgen-responsive prostate cancer LNCaP cells, five miRNAs, miR-32, miR-148a, miR-99a, miR-21, and miR-221, showed differential expressions in CRPC samples and in the presence of ARBSs upstream, with no other genes nearby [77]. In addition, all of them have been functionally validated to be directly transcriptionally regulated by AR using androgen or anti-AR drugs for treating androgen-sensitive PC cells, further confirming these miRNAs as the AR-regulated genes.

#### 2.1.1. miR-32

miR-32 is located on chromosome 9 in intron 14 of the c9orf5 gene. Jalava *et al.* identified an ARBS in the approximately 14 kb upstream of the miR-32 locus [77]. They also reported that miR-32 is overexpressed only in CRPC but not in primary untreated PC compared to benign prostatic hyperplasia (BPH) [77]. However, upregulation of miR-32 was found in localized PC [78], and its expression may predict poor outcomes after a prostatectomy [79], suggesting that miR-32 might not exclusively be involved in CRPC but in an early event of prostate carcinogenesis. The oncogenic function of miR-32 in PC was linked to its directly targeted gene, B-cell translocation gene 2 (*BTG2*) [77]. *BTG2* (also known as *PC3* or *TIS21*) is a tumor suppressor gene, which belongs to the antiproliferative (APRO) gene family. BTG2 expression is found to be decreased in a variety of human cancers and plays important roles in controlling cell cycle progression, cell differentiation, DNA damage repair, and apoptosis [80]. An immunohistochemical analysis in prostatectomy specimens demonstrated an association of low BTG2 protein expression with a higher clinical stage of prostate cancer and a shorter, cancer progression-free period. More strictly, CRPC cells showed significantly (*p* < 0.0001) lower staining intensities of BTG2 compared to untreated PC cells [77]. In addition to negatively regulating cell cycle progression in response to DNA damage and other stresses [81], the tumor-suppressor function of BTG2 in the prostate epithelium was also demonstrated by the downregulation of BTG2 expression in non-tumorigenic prostate cells, which caused prostate cell transformation through induction of the epithelial-mesenchymal transition (EMT) phenotype [82]. A more-recent study uncovered BTG2 as an AR-interacting protein in the repression of AR transcriptional activity through directly binding to the AR at an LxxLL motif [83]. This finding may provide evidence for a novel oncomiR function of miR-32 in modulating the AR signal pathway in CRPC via regulating AR transcriptional coregulators.

#### 2.1.2. miR-148a

miR-148a is located on chromosome 7p15.2. Two ARBSs has been identified in close proximity to miR-148a, one 72 kb upstream and the other 8.5 kb downstream [77]. Gene expression studies with microarray analysis revealed that miR-148a is upregulated in clinical prostate carcinoma compared to normal prostate tissues [84]. Comparison data for CRPC specimens have not been established. A study conducted with hormone-sensitive LNCaP cells supported the oncogenic function of miR-148a in PC, where it was shown to facilitate LNCaP cell growth by repressing expression of its target cullin-associated and neddylation-dissociated 1 (CAND1) [85]. However, contradictory data are also available regarding the expression level of miR-148a in PC. Using a series of PC cell lines, Fujita *et al.* found that expression level of miR-148a was lower in hormone-refractory PC3 and DU145 cells than in LNCaP hormone-sensitive PC cells and PrEC normal human prostate epithelial cells [86]. They also demonstrated that ectopic expression of miR-148a in PC3 cells inhibited cell growth, migration, and invasion, and potentiated paclitaxel-induced cytotoxicity. It is mediated by deregulation of MSK1 [86], a known serine/threonine kinase downstream of extracellular signal-regulated kinase (ERK) or p38 mitogen-activated protein kinase (MAPK) in response to environmental stimuli [87,88]. The contrasting behaviors of miR-148a in hormone-sensitive and hormone-refractory PC seen in the cell line system might not represent its performance in actual clinical settings. Further comparison data for CRPC specimens are needed to clarify the role of miR-148a in disease progression.

#### 2.1.3. miR-99a

miR-99a is located on chromosome 21 and encoded with let-7c and miR-125b-2 within an intron of the long non-coding RNA, *LINC00478*. Five ARBSs, ARBS1 and ARBS2 within 50 kb of the transcription start site (TSS) of the long transcript of the *LINC00478* gene and ARBS3, ARBS4, and ARBS5 within 50 kb of the TSS of the short variant, were identified by genome-wide ChIP-seq analyses. Of these, androgen-induced AR binding was only seen at ARBS1 and ARBS2, through which androgen represses the expression of a miR-99a/let7c/125b-2 cluster in AR-positive PC cells [89]. Studies using cell lines and human prostate tumor samples confirmed the underexpression of miR-99a in PC, and showed that the reduction in miR-99a provides a growth advantage for AR-positive PC cells under an androgen-depleted condition [89,90]. Among the androgen-induced target genes of the miR-99a, insulin-like growth factor 1 receptor (IGF1R) and mammalian target of rapamycin (mTOR) are key factors responsible for androgen-induced growth by downregulation of miR-99a [89,90]. Increased levels of the IGF1R are expressed in a majority of primary and metastatic PC cases, and upregulation of the IGF1R signaling axis was shown to drive the survival of PC cells in many studies [91,92,93]. Similarly, activation of the PI3K/AKT/mTOR pathway was strongly implicated in the development of ADT resistance of PC through a dynamic interplay between this pathway and AR signaling axes [94]. Preclinical data showed that targeting mTOR along with antiandrogen treatment exhibited additive antitumor effects in a prostate-specific Pten (pseudogene of the tumor suppressor)-deleted mouse model, supporting the rationale for combining AR and mTOR inhibitors to treat CRPC [95]. As downregulation of miR-99a requires an active AR, it is expected that the hyperactivated AR frequently seen in CRPC could trigger increased expressions of IGF1R and mTOR through transcriptional repression of miR-99a, which may contribute to CRPC progression.

#### 2.1.4. miR-21

miR-21 is located on chromosome 17q23.2, an area frequently associated with genomic amplification in several cancer types, including PC at the stage of hormone refractory and metastasis [96,97]. The promoter region of miR-21 contains a highly conserved androgen response element, and can be directly bound by the AR in response to androgen [98]. Besides androgen responsiveness that relies on the AR, miR-21 expression was also detected in several AR-negative PC cells [99], suggesting that both AR-dependent and AR-independent pathways contribute to elevated miR-21 expression in PC. In addition to the AR, two transcriptional factors, activator protein 1 (AP-1) and signal transducer and activator of transcription 3 (STAT3), can also directly activate miR-21 after PMA and interleukin (IL)-6 stimulation, respectively [100,101]. This is relevant because progression to CRPC is closely associated with high levels of AP-1 and STAT3 [102,103]; elevation of miR-21 in PC, especially CRPC, is therefore partially due to aberrant activation of AP-1 and STAT3. Clinical investigations revealed a significant association of higher expression levels of miR-21 with advanced clinicopathological features and poor prognoses in patients with PC [77,104,105,106]. Overexpression of miR-21 alone is sufficient for androgen-dependent tumors to overcome castration and become androgen independent in a human prostate cancer xenograft model [98]. These observations strongly support the action of miR-21 as an oncomiR in promoting the progression and recurrence of CRPC. Several target genes of miR-21, such as *PDCD4*, *BMPR2*, *ANP32A* and *MARCKS*, exhibit tumor-suppressor activities to reduce cell motility, inhibit invasiveness, promote apoptosis, and induce cell cycle arrest in PC cells [107].

#### 2.1.5. miR-221

miR-221 is encoded with miR-222 by a gene cluster on the X chromosome. Unlike many AR-responsive genes which are upregulated by AR binding in the presence of androgen, the expression level of miR-221/-222 in prostate cancer is suppressed by androgen [77]. The differential expression pattern of miRNA-221 associated with PC progression remains contradictory. Two independent cohort studies using microarray and real-time reverse-transcription polymerase chain reaction (RT-PCR) technology revealed a progressive downregulation of miR-221 in aggressive PC, with associations with the Gleason score, clinical recurrence, and/or metastasis [108,109]. However, Li *et al.* using *in situ* hybridization in a tissue microarray containing 169 radical prostatectomy tissue samples demonstrated positive associations of miR-221 expression with the pathological stage, lymph node metastasis, capsular invasion, organ-confined disease, Gleason score, biochemical recurrence, and patient follow-up [104]. Several studies using well-established cell lines or clinically relevant patient-derived primary cell lines also reported that the expression of miR-221 was higher in androgen-independent PC cells than in androgen-dependent cells, implying a role of miR-211 in CRPC development [110,111,112]. In a CRPC progression cell model consisting of the androgen-dependent LNCaP and its derived CRPC cell line, LNCaP-Abl, miR-221 was found to be significantly upregulated in LNCaP-Abl compared to LNCaP cells, and its ectopic overexpression in LNCaP triggered CRPC phenotypes, including reduced expressions of androgen-responsive genes and an increase of androgen-independent growth [113]. An extended mechanistic study identified two miR-221 targets, *HECTD2* (HECT domain containing E3 ubiquitin protein ligase 2) and *RAB1A* (member RAS oncogene family), the downregulation of which resulted in reactivation of AR signaling and activation of new cyclins, leading to the development or maintenance of the CRPC phenotype [114]. Meanwhile, miR-221 can induce the neuroendocrine phenotype of PC cells by targeting *DVL2*, a key mediator of the Wnt signaling pathway, to drive metastatic CRPC [115].

### 2.2. miRNAs Regulating AR Expression (Andro-miRs)

As AR mRNA contains a long 3′-UTR, it is very likely that AR is targeted by a range of miRNAs. To systematically characterize putative miRNAs which are able to regulate this long AR 3′-UTR, Östling *et al.* conducted miRNA gain-of function screen in a panel of human PC cell lines and defined 71 unique miRNAs that affected AR protein expression [116]. Among these miRNAs, miR-135b, miR-185, miR-297, miR-299-3p, miR-34a, miR-34c, miR-371-3p, miR-421, miR-449a, miR-449b, miR-634, miR-654-5p, and miR-9 were characterized as andro-miRs based on their ability to suppress AR expression by directly binding to target sequences in AR 3′-UTR [116]. Functional assays clearly demonstrated that downregulation of the biological activities of these andro-miRs of the AR and subsequent AR-targeted gene expressions in PC cells lead to decreased androgen-induced proliferation and increased cellular apoptosis. However, of these, only levels of miR-34a and miR-34b were found to be inversely correlated with AR protein levels in human PC tissue specimens [116,117], and they were linked to aggressiveness, the WHO grade, PSA levels, and the occurrence of metastases [118]. Other studies using computational analyses also identified a potential target site for miR-488* [119] and miR-205 [120] in the 3′-UTR of AR mRNA. In addition, some miRNAs such as let-7c [121] antagonize AR expression as evidenced by an indirect targeting mechanism.

#### 2.2.1. miR-34a

The miR-34 family consists of miR-34a, miR-34b, and miR-34c at two genomic loci. miR-34a is encoded by its own transcript from chromosome 1p16, whereas miR-34b and miR-34c are co-transcribed from chromosome 11q23. Studies showed that expression of miR-34a is reduced or lost in many cancers such as ovarian, leukemia, pancreatic, colon, and prostate cancers [122]. Loss of p53 is expected to reduce miR-34a expression, which is implicated in the negative control of the cell cycle, senescence, and apoptosis [123,124]. In PC, miR-34a expression appears to be epigenetically silenced due to hyper-methylation of the miR-34a promoter [117]. Enforced expression of miR-34a in PC cells resulted in reduced expressions of the AR, PSA, and Notch-1 concomitant with inhibition of the self-renewal capacity [125], a phenotype associated with cancer stem cells. Interestingly, the cell adhesion and stem cell marker, CD44, was discovered as a direct target gene for miR-34a. Forced expression of miR-34a reduced clonogenic growth, tumor regeneration and metastasis of CD44^+^ PC cells. Conversely, inhibition of miR-34a by antagomiRs potentiated tumor development and metastasis of CD44^−^ PC cells [126]. Collectively, these findings possibly imply that miR-34a is also an important regulator of cancer stem cell properties as the CRPC phenotype in human PC patients.

#### 2.2.2. miR-205

miR-205 levels in CRPC were found to be significantly lower than in hormone-naive patients, and to have an inverse correlation with AR levels in malignant epithelial cells, but there was no correlation in benign epithelium [120]. A pathway analysis of genes directly regulated by miR-205 revealed that some genes, such as *IL-8* and *EDN1*, are known to enhance expression of the AR, and others are enriched in the MAPK/ERK, mTOR, and IL-6 signaling pathways. AR activation by IL-6 is well documented [127,128]. In addition, phosphorylation of the AR by MAPK helps recruit AR co-regulators and results in hyper-sensitization to the castrated level of androgen [129,130]. Targeting AKT/mTOR and ERK MAPK signaling using pharmaceutical inhibitors showed therapeutic efficacy for CRPC in a preclinical mouse model [131]. Boll *et al.* recently demonstrated that miR-205, miR-203, and miR-130a are jointly downregulated in PC; reconstitution of miR-205 in androgen-dependent PC cells resemble the effect of androgen deprivation, including changes in cell morphology and impeded growth by suppressing several AR coactivators, including CDK1, PSAP, PSMC3IP, and PARK7 [132]. Although it remains unknown whether reduction of miR-205 alone is sufficient to drive CRPC, these results all point to a decrease in miR-205 in PC increasing the ligand-independent activation of the AR. Additional attention to the relationship between miR-205 and PC progression was focused on its antimetastatic role in concurrent repression of a cohort of pro-metastatic genes, including *N*-chimaerin, *ErbB3*, *E2F1*, *E2F5*, *ZEB2*, and protein kinase Cε which are known to drive the EMT [133]. In conjunction with the finding that restoring the expression of miR-205 impaired cell growth, migration, clonability, and invasive properties of PC cells [134], miR-205 might possibly account for part of the increased risk of metastasis that is seen in CRPC patients.

#### 2.2.3. miR-488*

miR-488* is another miRNA that was found to directly target the AR. Sikand *et al.* recently identified a target site for miR-488* in a region between nucleotides 4266 and 4289 of the AR 3′-UTR [119]. Although miR-488* expression was not detected in PC cell lines irrespective of their androgen dependence and no evidence of changes in human PC specimens, ectopic expression miR-488* can effectively suppress AR protein expression, inhibit the AR target gene PSA expression in an androgen-dependent manner, retard the growth, and induce apoptosis of PC cells [119]. These findings thereby suggest a tumor-suppressor role of miR-488* in disrupting the AR signaling axis by deregulating AR expression.

#### 2.2.4. miR-124

miR-124 is recognized as a tumor suppressor involved in the control of multiple steps of malignant processes, including tumor cell proliferation, invasion, angiogenesis, and metastasis in many cancer types [135,136,137,138,139,140,141]. Sufficient evidence indicated that loss of miR-124 expression in malignant prostatic cells is epigenetically regulated by promoter hypermethylation, and it is associated with elevated AR levels in both cell lines and clinical prostate samples [135,138,142]. Shi *et al.* recently identified an miR-124-binding site in the first 436 bases of the AR 3′-UTR, validating the action of miR-124 in the negative regulation of the AR [135]. Directly targeting the AR by miR-124 subsequently induces upregulation of p53, leading to growth inhibition and apoptosis of AR-positive PC cells and xenograft tumors [135]. As the AR regulates miR-125b, and miR-125b directly targets p53 [143], miR-124 upregulation of the expression of p53 may due, at least in part, to the miR-124/AR/miR-125b signaling pathway. Alternatively, miR-124 induction of the p53 pathway may be mediated by another target gene, *HMGA* [135], which has the ability to bind and inactivate p53’s function [144]. Interestingly, while the AR is the target gene of miR-124, the AR can positively regulate miR-124 expression via inducing androgen, suggesting a negative feedback loop between the AR and miR-124 controlling the progression of CRPC [142].

#### 2.2.5. Let-7c

Let-7 was first discovered and has been well studied in *Caenorhabditis elegans* [145]. In humans, the let-7 family consists of let-7a, let-7b, let-7c, let-7d, let-7e, let-7f, let-7g, let-7i, miR-98, and miR-202 [146], and is commonly viewed as tumor suppressors linked to tumor progression in many cancers as well as poor patient prognoses. Let-7c was found to be inversely correlated with the AR in cell culture, xenografts of prostate mouse models, and human PC specimens, whereas the expression of Lin28, a master regulator of let-7 miRNA processing, is correlated positively with AR expression. More importantly, let-7c antagonizes AR transcription by targeting c-Myc expression [121], one of the transcriptional factors binding to the AR promoter [147]. On the other hand, Gao *et al.* showed that the *c-myc* gene enhancer contains an AR-binding site by ChIP assays, and confirmed that AR serves as a positive regulator for *c-myc* transcription, which is ligand-independent [148]. Indeed, both the AR and c-Myc are commonly increased in human CRPC tumor progression [148,149]. Suppression of the AR and c-Myc resulted in a growth retardation of PC cells, and ectopic expression of c-Myc attenuated the anti-growth effects of AR suppression in an androgen-independent manner, indicating a cooperative survival signal pathway between the AR and c-Myc that promotes PC growth [148]. However, how let-7c negatively regulates c-Myc expression in PC is not fully understood. A bioinformatic analysis of potential binding sites for miRNAs in the UTRs of a set of human oncogenes predicted an interaction between let-7c and *c-myc*, and it was confirmed by experimental validation [150], proving that *c-myc* is a let-7c target gene. An RNA-binding protein, myo-inositol monophosphatase 1 (IMP1), which is known as a direct let-7 target was demonstrated to specifically recognize and regulate c-Myc expression [151,152], providing another hypothetical mechanism for let-7c in the transcriptional modulation of c-Myc and sequential AR dysregulation. Taken together, loss of let-7c in PC cells might sensitize the AR signaling pathway to lower levels of androgens (ligand-dependent activation) and/or the c-Myc-mediated oncogenic pathway (ligand-independent cross-talk).

## 3. miRNAs in Prostate Cancer (PC) Metabolism

**Figure 2:**
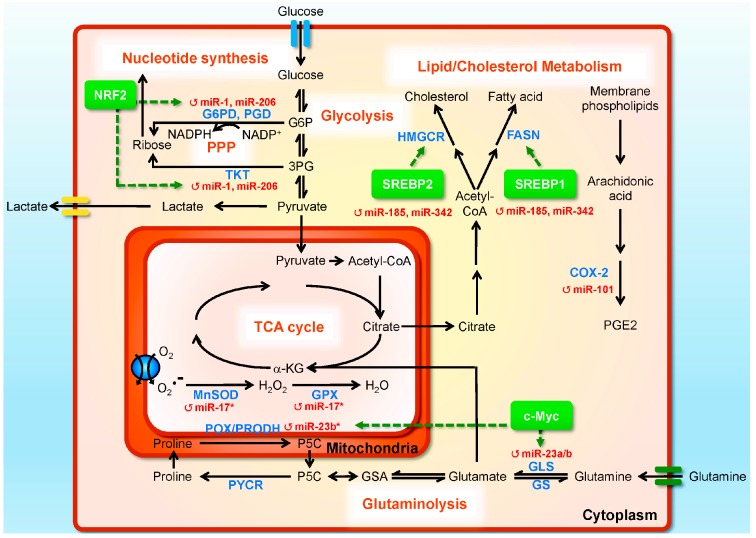
The crucial role of miRNAs in coordinating vast prostate cancer (PC) metabolic processes. A number of miRNAs can reprogram PC metabolism by fine-tuning expressions of metabolic enzymes, and their regulators and signaling pathways. The steps affected by miRNAs are labelled by red circular arrows. Metabolites, metabolic enzymes, and transcription factors are indicated in black, blue, and green, respectively. All of the metabolic pathways are labeled with orange in bold. Black arrows indicate the direction of metabolite conversion, whereas green arrows point out the miRNAs or metabolic enzymes affected by the transcription factors. 3PG, 3-phosphoglyceric acid; α-KG, α-ketoglutarate; COX-2, cyclooxygenase-2; Δ1-pyyroline-5-carboxylate; FASN, fatty acid synthase; G6P, glucose-6-phosphate; G6PD, glucose-6-phosphate dehydrogenase; GLS, glutaminase; GPX2, glutathione peroxidase-2; GS, glutamine synthetase; GSA, glutamic-γ-semialdehyde; HMGCR, 3-hydroxy-3-methylglutaryl CoA reductase; MnSOD, manganese superoxide dismutase; NRF2, nuclear factor erythroid-2-related factor 2; P5C, Δ1-pyyroline-5-carboxylate; PGD, 6-phosphogluconate dehydrogenase; PGE2, prostaglandin E2; POX/PRODH, proline oxidase/proline dehydrogenase; PPARA, peroxisome proliferator-activated receptor α; PPP, pentose phosphate pathway; PYCR, P5C reductase; SREBP-1, sterol regulatory element binding protein 1; SREBP-2, sterol regulatory element binding protein 2; TCA, tricarboxylic acid; TKT, transketolase; TrxR2, thioredoxin reductase-2.

Altered cellular metabolism is a classical feature of tumors. These metabolic shifts deliberate several biological benefits to cancer cells, including the elevation of biosynthesis, adenosine triphosphate (ATP) generation, detoxification, and adaptation to adverse environments, and thereby help cancer cells to rapidly proliferate and invade [153]. Oncogene and tumor suppressors, the discoveries of which revolutionized cancer research, are now linked to metabolism regulation, connecting genetic alterations in cancers to their metabolic phenotypes. These findings make cancer metabolism once again an intensely studied area of tumor biology. Interestingly, the scenario of metabolic shift in PC cells seems to be much more unique and complicated. The prostate gland has a unique specialized metabolic role as a secretor of prostatic fluid, which contains very high levels of citrate to maintaining sperm viability. To accumulate citrate for secretion, the normal metabolic activity of luminal epithelial cells is altered to inhibit cis-aconitase within the tricarboxylic acid (TCA) cycle. This impaired efficiency of the TCA cycle suggested that the basal energy metabolism in the untransformed prostate gland is more glycolytic and associated with biomass production [154,155]. Unlike normal prostate cells, PC cells could oxidize citrate in the TCA cycle, leading to decrease in citrate level. In addition, other metabolites, such as spermine, phosphocholine, sarcosine, lactate, taurine, glutamate, lysine, myo-inositol, and ω-6-fatty acids, have been reported to be present at significantly higher levels in PC tissues [155]. Furthermore, recent studies have provided the systematic evidence that AR signaling could influence metabolism and biosynthesis at multiple key regulatory steps [40,154]. Given the relevance of miRNAs to tumor progression, it is not surprising that studies identifying miRNA-mediated metabolic regulation have also intensified. To date, numerous of miRNAs have been shown to regulate tumor metabolism, either through directly targeting crucial enzymes/transporters of metabolic pathways or by indirectly modulating transcription factors and complex signaling networks [156,157,158]. Here, we briefly summarize recent findings pertaining to miRNA-mediated metabolic reprogramming in PC (Table 1; Figure 2).

**Table 1:** Summary of miRNA regulation in prostate cancer metabolism.

miRNA	Target Gene	Up/Downregulation in PC	Metabolic Activity/Signaling Pathway	Tissues/Cell Lines	References
Glucose metabolism					
miR-1, miR-206	*G6PD*, *TKT*, *PGD*, *GPD2*	↓	NRF2 signaling; reprogramming glucose metabolism by directing carbon flux toward the PPP and the TCA cycle	DU145 prostate cancer and A549 lung carcinoma cell lines	[159]
Lipid metabolism					
miR-185,miR-342	*SREBP-1, SREBP-2*	↓	SREBP signaling; lipogenesis and cholesterogenesis	LNCaP and C4-2B prostate cancer cells	[160]
miR-17/92	*PPARA*	↑	PPARA signaling; lipogenesis	LNCaP prostate cancer cells	[161]
miR101	*COX-2*	↓	COX-2/PGE2 pathway	BPH1, LNCaP, and PC3 prostate cancer cells, gastric cancer, endometrial serous adenocarcinoma, colon cancer	[162,163,164,165,166]
Glutamine metabolism					
miR-23a/b	*GLS*	↓	c-Myc signaling; glutamine metabolism	Burkitt lymphoma cells, P-493 and PC3 prostate cancer cells	[167]
miR-23b-3p	*POX/PRODH*	↑	c-Myc signaling; proline and glutamine metabolism	Burkitt lymphoma cells, P-493 and PC3 prostate cancer cells	[168]
Mitochondrial antioxidant metabolism					
miR-17-3p	*MnSOD*, *GPX2*, *TrxR2*	↓	Mitochondrial antioxidant	PC3 prostate cancer cells	[169]

COX-2, cyclooxygenase-2; G6PD, glucose-6-phosphate dehydrogenase; GLS, glutaminase; GPD2, glycerol-3-phosphate dehydrogenase; GPX2, glutathione peroxidase-2; MnSOD, manganese superoxide dismutase; NRF2,nuclear factor erythroid-2-related factor 2; PC, prostate cancer; PGD, phosphogluconate dehydrogenase; PGE2, prostaglandin E2; POX/PRODH, proline oxidase/proline dehydrogenase; PPARA, peroxisome proliferator-activated receptor α; PPP, pentose phosphate pathway; TCA, tricarboxylic acid; SREBP-1,sterol regulatory element binding protein 1; SREBP-2, sterol regulatory element binding protein 2; TKT, transketolase; TrxR2, thioredoxin reductase-2; ↓, downregulation; ↑, upregulation.

### 3.1. miRNAs Regulating Glucose Metabolism

Cancer cells prefer using aerobic glycolysis to convert available glucose (the Warburg effect) thereby diverting glycolic intermediates to various anabolic processes. For instance, one of the major glucose catabolism pathways, the pentose phosphate pathway (PPP), guides glucose flux to the oxidative branch and supplies cells with nucleotides and nicotinamide adenine dinucleotide phosphate (NADPH; an essential reducing agent important for redox homeostasis) for ribose biogenesis, detoxification of intracellular reactive oxygen species (ROS) and reductive biosynthesis. Meanwhile, in rapidly proliferating cancer cells, most carbon moving in the tricarboxylic acid (TCA) cycle is directed to biosynthetic pathways, leading to the constant efflux of intermediates and the synthesis of lipids required for organelle and membrane assembly.

A report by Singh *et al.* demonstrated that in PC cells, PPP activity is controlled by the miRNAs, miR-1 and miR-206 [159]. With the same seed sequence, miR-1 and its identical paralog, miR-206, bind to the same targets, including three key genes (glucose-6-phosphate dehydrogenase, *G6PD*; phosphogluconate dehydrogenase, *PGD*; and transketolase, *TKT*) of the PPP and one gene (glycerol-3-phosphate dehydrogenase, *GPD2*) associated with the TCA cycle and carbohydrate/lipid metabolism [159]. Notably, the transcription factor, nuclear factor erythroid-2-related factor 2 (NRF2), attenuates of expressions of these two miRNA species, which in turn upregulates PPP enzymes and reprograms glucose metabolism [159]. Therefore, this NRF2-mediated metabolic alteration promotes tumor growth by providing cancer cells with building blocks for nucleotide synthesis, as well as NADPH for redox homeostasis, all of which are required for accelerated proliferation. These findings reflect a novel link among miRNA regulation, gain of NRF2 function, and glucose metabolism in PC.

### 3.2. miRNAs Regulating Lipid Metabolism

Lipids are a group of water-insoluble, organic compounds including fats, triglycerides, phospholipids, sterols, sphingolipids, and others. The main biological functions of these molecules include energy storage, signaling, and surfing as structural components of cell membranes. Accumulating evidence demonstrates that the lipid metabolism of cancer cells is reprogrammed to cope with different needs for energy storage, cell membrane components, and activation of signaling transduction. Notably, aberrant lipid and cholesterol metabolism is tightly associated with PC development and progression to end-stage disease [170].

The regulation of cholesterol/fatty acid/phospholipid biosynthesis is mediated by several crucial transcription factors, such as sterol regulatory element-binding proteins (SREBPs). SREBP-1 controls genes involved in the biosynthesis of fatty acids, lipids, and cholesterol, while SREBP-2 more specifically modulates cholesterol metabolism and homeostasis. In PC cells, two miRNAs, miR-185 and 342, were identified to regulate the biosynthesis of lipid and cholesterol by inhibiting SREBP-1 and -2 expressions, thereby downregulating their target genes, fatty acid synthase (FASN) and 3-hydroxy-3-methylglutaryl CoA reductase (HMGCR) [160]. Coincident with their inhibition of lipogenesis and cholesterogenesis, overexpression of these two miRNA species suppressed cell growth, migration, and invasion, thus revealing the tumor-suppressor roles of miR-185 and -342. Those studies suggest that interference by abnormal lipogenesis and cholesterogenesis may provide potential therapeutic approaches for treating prostatic malignancy [160].

In addition to SREBPs, the nuclear transcription factor, peroxisome proliferator-activated receptor α (PPARA), is another major regulator that modulates lipid metabolism. PPARA was predicted to be a target of the miR-17/92 cluster [161]. In PC cells, testosterone and 1,25-dihydroxyvitamin D3 (1,25(OH)_2_D_3_)-induced miR-17/92 downregulation was reported to alleviate the miRNA-mediated inhibitory effect on PPARA mRNA, thereby increasing PPARA transcript stability, promoting neutral lipid synthesis, and retarding tumor progression [161]. Those findings not only suggest that testosterone- and 1,25(OH)_2_D_3_-mediated signaling can switch energy homeostasis from lipolysis (energy production) to lipogenesis (energy storage), but also provide a mechanistic explanation for epidemiological analysis connecting the increasing incidence of PC with declining serum testosterone and low 1,25(OH)_2_D_3_ levels [161].

Another group of hormone-like lipid compounds, the prostaglandins (PGs), participate in a variety of biological functions to sustain homeostasis and mediate inflammatory responses. Cyclooxygenase-2 (COX-2; also called prostaglandin synthase-2) is the central, rate-limiting, and inducible enzyme for catalyzing the biosynthesis of PGs from arachidonic acid. COX-2 was reported to be constitutively elevated in a variety of malignancies including prostate carcinoma and to contribute to tumor progression. Recent studies showed that miR-101 is able to target COX-2 in various cancers [162,163,165,166]. Similar observations were made in PC cells [163]. Ectopic expression of miR-101 suppresses COX-2 protein expression, resulting in repression of COX-2-associated cell growth factors, cell proliferation, and tumor growth [163]. Along these lines, blockage of the COX-2 pathway by exogenous miR-101 may provide an alternative cancer therapeutic strategy.

### 3.3. miRNAs Regulating Glutamine Metabolism

Apart from glucose, increasing evidence suggests that glutamine is another crucial molecule in tumor growth and progression. Given the tight dependence of many cancer cells on this amino acid, the adaptive accelerated and preferential glutamine metabolism (glutaminolysis) by tumor cells seems to provide reagents for increased lipogenesis and nucleic acid biosynthesis during rapid proliferation. Glutaminase (GLS)-catalyzed deamination converts glutamine to glutamate. Glutamate is an essential component of glutathione. Meanwhile, after conversion to α-ketoglutarate (α-KG), glutamate can serve as an central energy source through anaplerotic input into the TCA cycle, linking glutaminolysis to the biosyntheses of proteins, nucleotides, and lipids as well as redox homeostasis and energy metabolism. Currently, glutaminolysis is reported to be controlled by several miRNAs [171]. In PC and B lymphoma cells, mitochondrial glutaminase was reported to be a direct target of miR-23a and miR-23b [167]. Interestingly, c-Myc, the well-known oncogenic transcription factor frequently upregulated in different tumors to stimulate cell proliferation and regulate microRNAs, transcriptionally represses miR-23a/b, thereby restoring glutaminase expression and promoting glutamine catabolism. This pathway provides a novel link among c-Myc-mediated regulation of miRNAs, glutamine metabolism, and energy/redox homeostasis in PC [167].

The significance of glutamine catabolism in PC metabolism was further highlighted by another report. It is less well recognized that glutamate can also be sequentially converted to proline, while proline can also be converted to glutamate through proline catabolism. Proline oxidase (POX; also named as proline dehydrogenase; PRODH), the first step in proline catabolism, is a mitochondrial inner membrane enzyme and a tumor suppressor that impedes proliferation and induces apoptosis. Interestingly, processed from the same transcript as miR-23b, miR-23b* (miR-23-5p) was found to mediate POX/PRODH downregulation in PC cells [168]. Also noted, c-Myc suppressed POX/PRODH expression primarily by upregulating miR-23b* [168]. Together, the metabolic connection between proline and glutamine conferred by c-Myc underlines the complexity of PC metabolism, and further supports the integral role of c-Myc in glutamine metabolic processes and tumorigenesis.

### 3.4. miRNAs Regulating Mitochondrial Oxidative Metabolism

A crucial consequence of oxygen metabolism is the generation of reactive oxygen species (ROS). Due to their accelerated proliferation and metabolism, cancer cells require high levels of antioxidant proteins to remove the rapid generation of ROS [172]. Manganese superoxide dismutase (MnSOD), glutathione-dependent peroxidase (GPX), and thioredoxin-dependent peroxidase (TrxR2), are the primary defense system in mitochondria and essential for the detoxification of ROS. In PC, miR-17* (miR-17-3p) was demonstrated to function as a negative regulator for these enzymes [169], linking an accumulation of ROS with the suppression of tumorigenecity. Together, these findings propose therapeutic alternatives against tumor growth by inhibiting mitochondrial defense capacities [169].

Collectively, our current understanding about miRNA-mediated metabolic regulation in PC clearly still remains far from complete, but the evolving concept of miRNAs as regulatory modules that cooperate with diverse signaling and metabolic pathways may expand the repertories of AR signaling and AR-mediated metabolic regulation. Remarkably, several miRNA-mediated metabolic regulations identified in PC could also be found in other cancer types. These consistent findings in various cancer types not only strengthen metabolic alterations as a hallmark of cancer, but also reveal their relevance in the overall tumorigenesis and transformation processes. Obviously, identification of the potential miRNAs targeting PC-specific metabolic alterations will be of great clinical significance. Given the complex interplay among different metabolic pathways, AR responsiveness, AR signaling status, PC types and stages, it will be necessary to characterize PC metabolome in large sample cohorts to verify and validate potential miRNAs involved in metabolic shifts during CRPC development.

## 4. Long ncRNAs (lncRNAs) in Castration-Resistant Prostate Cancer (CRPC)

With advances in sequencing technology and updated annotations available for lncRNAs, many lncRNAs associated with PC have been identified and the number continues to grow [173,174,175,176]. Alterations of cancer-related lncRNAs include aberrant expression and SNPs that display oncogenic or tumor-suppressive properties. Genome-wide association studies revealed that a large proportion of genetic variations associated with PC are enriched in lncRNA regions, suggesting that these lncRNAs are functionally linked to cancer risk [177,178]. The intergenic 8q24 locus, for instance, is the major PC susceptibility region which transcribes SNP-containing RNA molecules that have the capacity to promote hormone-dependent PC into highly maliganant CRPC [179]. It is possible that the risk-related SNPs on 8q24 may control expressions of the encompassed lncRNAs, PCAT1, PRNCR1, and PVT1, in PC cells and further contribute to cancer progression [34,180]. It was demonstrated that PVT1 expression was correlated with the risk-related variant rs378854 [181], and PVT1 expression is required to maintain Myc protein levels and the oncogenic potential of Myc-driven cancer [182]. While correlations of 8q24 SNPs with PCAT1 and PRNCR1 expressions have not been described, overexpression of these lncRNAs by PC cells is implicated in cell viablilty and proliferation [173,183,184]. Similar to the 8q24 locus, an SNP located on lncRNA AC1127096 at 19q13 was also reported to be associated with a high risk of PC [184,185]. Polymorphisms on the highly cancer-associated lncRNA PCGEM1 was also suggested to contribute to cancer risk in Chinese men [186].

While our understanding of expression regulation of lncRNAs remains limited, most lncRNAs identified in PC appear to be androgen responsive, and many of them are downstream targets of the AR (Table 2). This is understandable, as aberrant AR signaling and activity play central roles in PC progression. Dysregulation of these androgen-responsive lncRNAs, in turn, gives rise to tumorigenesis via various AR-dependent mechanisms. In addition to the AR, transctiptional factors such as E2F1 and estrogen receptor ERα, which can drive androgen-independent growth, were also found to respectively regulate the lncRNAs, ANRIL and NEAT1 [187,188]. The major molecular mechanism of lncRNAs that contribute to CRPC thus far discovered is discussed below.

**Table 2:** Prostate cancer-associated long non-coding RNAs.

lncRNA	Function	Regulation	Alteration in PC	Oncogene/Tumor Suppressor	Clinical Association	References
ANRIL	Functions in the DNA damage response. Represses INK4a-INK4b-ARF by binding with PRC2.	Upregulated by E2F1	Overexpression	Oncogene	–	[188]
CTBP1-AS	Promotes AR transactivity by repressing the co-repressor CTBP1. Promotes both hormone-dependent and castration-resistant growth.	Androgen responsive	Overexpression	–	–	[189]
DRAIC (LOC145837)	Suppresses cellular transformation, migration and invasion.	Repressed by AR	Downregulated in CRPC	Tumor suppressor	Prognostic marker	[190]
GAS5	Induces apoptosis.	Self regulation	Downregulated in CRPC cells	–	–	[191,192]
H19	Suppresses metastasis and cell migration. Enhances stemness by regulating Oct4 and Sox2 expression.	–	Downregulated in metastatic cell lines	Tumor suppressor	–	[193,194]
HOTAIR	Promotes cell proliferation, migration, invasion, and survival.	–	Overexpressed in CRPC cells	Oncogene	–	[195,196]
	Forms a complex with PRC2 and suppresses AR transcription.					
Linc00963	Promotes cell proliferation, migration, and invasion.	–	Overexpressed in CRPC cells	Oncogene	–	[197]
MALAT-1	Promotes cell proliferation, migration, invasion, and survival.	–	Overexpression	Oncogene	Correlates with high Gleason scores, tumor stages, and CRPC	[175,198,199]
NEAT1	Promotes tumor proliferation through the ERα signaling pathway	Upregulated by ERα	Overexpression	Oncogene	Associated with progression	[187]
PCA3	Regulates AR signaling and cell proliferation	Androgen responsive	Overexpression	Oncogene	Diagnostic predictor of malignant patients	[200,201,202]
PCAT1	Represses BRCA2 and homologous recombination, and inhibits DNA repair. Promotes proliferation through stabilization of Myc mRNA.	–	Overexpression	Oncogene	–	[173,183,203]
PCAT18	Promotes cell proliferation, migration and invasion.	Upregulated by AR	Overexpressed in metastatic clinical specimens	Oncogene	–	[204]
PCAT29	Suppresses cell migration and metastasis.	Downregulated by androgen and the AR	Downregulated in CRPC	Tumor suppressor	Low expression correlated with poor prognostic outcomes	[190,205]
PCGEM1	Promotes cell proliferation, migration, invasion, and colony formation. Inhibits doxorubicin-induced apoptosis by attenuating p53 and p21.	Androgen dependent, upregulated by the AR; Regulated by cholesterols	Overexpressing SNP	Oncogene	Associated with high risk	[180,186,206,207,208,209,210,211,212]
	Enhances ligand-dependent and -independent AR activation through looping.					
	Regulates tumor metabolism by enhancing Myc transactivity.					
PlncRNA-1	Regulates cell proliferation and apoptosis. Regulates AR mRNA.	Upregulated by the AR	Overexpression	Oncogene	–	[213]
PRNCR1 (PCAT8)	Enhances ligand-dependent and -independent AR activation through looping.	–	Overexpressing SNP	Oncogene	–	[184,208]
SChLAP1	Promotes invasion and metastasis. Antagonizes the SWI/SNF complex.	–	Overexpression	Oncogene	Associated with progression and poor outcomes	[214,215]
TRPM2-AS	Regulates cellular responses to oxidative stress by controlling TRPM2 expression.	–	Overexpression	–	Associated with poor clinical outcomes and Gleason scores	[216]

lncRNA, long non-coding RNA; AR, androgen receptor; CRPC, castration-resistant prostate cancer; PSA, prostate-specific antigen; ERα, estrogen receptor α; PC, prostate cancer; SNP, single nucleotide polymorphism; ANRIL, antisense non-coding RNA in the INK4 locus; CTBP1-AS, CTBP1 Antisense RNA; DRAIC, downregulated RNA in cancer; GAS5, growth arrest-specific 5; H19, H19 imprinted maternally expressed transcript; HOTAIR, HOX transcript antisense RNA; MALAT-1, metastasis-associated lung adenocarcinoma transcript-1;NEAT1, nuclear enriched abundant transcript 1; PCA3, prostate cancer antigen 3; PCAT1, prostate cancer associated transcript 1; PCAT18, prostate cancer associated transcript 18; PCAT29, prostate cancer associated transcript 29; PCGEM1, prostate cancer gene expression marker 1; PRNCR1, prostate cancer noncoding RNA1; SCHLAP1, second chromosome locus associated with prostate-1; SWI/SNF, switch/sucrose non-fermentin; TRPM2-AS, TRPM2 antisense RNA.

### 4.1. lncRNAs in AR-Dependent Oncogenicity

Many lncRNAs associated with PC play oncogenic roles by modulating AR activity through various mechanisms. A study by Yang *et al* uncovered a sophisticated chromatin looping mechanism for AR transactivation mediated by two lncRNAs, PRNCR1 and PCGEM1. They demonstrated that by binding to the AR in the enhancer region, PRNCR1 recruits DOT1L methyltransferase to catalyze methylation of the AR on K349, which further mediates AR-PCGEM1 association and Pygo2 recruitment, thereby enhancing selective looping of the enhancer region to promoter and induce transcription of the target gene [208]. Interestingly, PRNCR1- and PCGEM1-mediated looping enhances oncogenic programming through both ligand-dependent and -independent AR activation. Knockdown of these lncRNAs in the CRPC cell line, CWR22Rv1, that expresses an AR splice variant, inhibits its xenograft growth *in vivo* [208]. This finding suggests possible roles of PRNCR1 and PCGEM1 in promoting advanced CRPC by enhancing ligand-independent AR activation. However, contrary views were raised pointing out the failure to reproduce physical interactions between these lncRNAs and AR, the lack of an association of these lncRNAs with poor disease outcomes, and unchanged levels of these lncRNAs in metastasized cancer [207,210]. Despite these conflicts and concerns, PCGEM1 and PRNCR1’s oncogenic roles in AR signaling are supported by other reports that reproducibly demonstrated the responsiveness of PCGEM1 upon androgen treatment [207,212], the binding and coactivating ability of PCGEM1 towards the AR [211], and evidence showing PRNCR1’s role in AR transactivation [184]. The association of PCGEM1 with prostate oncogenesis, in particular, is supported by its overexpression in prostate tumors that was repeatedly observed [174,207,209,210], and by its function in enhancing cancer cell survival, proliferation, invasion, and metabolic regulation [205,206,209,211]. Together, although PRNCR1 and PCGEM1’s association with metastatic CRPC remains inconclusive, the evidence collectively suggests their oncogenic properties in PC through the AR. Similar to PRNCR1 and PCGEM1, yet through a different mechanism, CTBP1-AS enhances AR transcriptional activity and promotes both hormone-dependent and castration-resistant tumor growth by repressing the AR-corepressor, CTBP1. CTBP1-AS also represses expression of the tumor suppressors, p53 and SMAD3 [189].

In addition to regulating AR transcriptional activity, lncRNA-mediated modulation of AR transcripts was also reported. Knockdown of PlncRNA-1 results in a decrease in AR mRNA and AR target genes, leading to growth retardation and apoptosis in both androgen-dependent and -indepemdent cell lines [213]. On the other hand, in a mast cell-infiltrated prostate tumor region, the HOTAIR-polycomb repressive complex supresses AR transcription, consequentely promoting cell invasion with increased matrix metalloproteinase-9 (MMP9) expression [195]. Finally, although the mechanism is unclear, androgen-responsive PCA3 was also reported to be involved in regulating AR signaling and cell proliferation. Knockdown of PCA3 reduces AR target gene expression and results in cell cycle arrest [201].

### 4.2. lncRNAs in Metabolic Regulation and Stress Responses

Metabolic alterations of cancer cells are critical for cells to sustain rapid proliferation and adapt to the dynamic tumor microenvironment. We previously reported that lncRNA PCGEM1 is a metabolic regulator in PC cells that promotes cell growth through upregulating aerobic glycolysis (the Warburg effect), the PPP, and lipid and glutamine metabolism [211]. These metabolic regulatory roles of PCGEM1 appear to be independent of the presence of androgen or the AR, and are mediated via Myc activation. PCGEM1 physically associates with Myc, enhances Myc recruitment to its target promoters, and enhances its transactivation. Although associations of PCGEM1 with CRPC and metastasis remain inconclusive (as discussed in the previous section), PCGEM1’s role in modulating tumor metabolism in an AR- and hormone-independent manner suggests the possibility that PCGEM1-mediated metabolic regulation likely contributes to early cellular adaptation upon androgen ablation. In addition to PCGEM1, lincRNA-p21 was also revealed to be an important regulator of the Warburg effect in cancer cells, essential for hypoxia-enhanced glycolysis and tumor cell growth [217]. Under hypoxia, hypoxia-inducible factor (HIF)-1α induces lincRNA-p21 expression which in turn, associates with HIF-1α and promotes HIF-1α accumulation by attenuating VHL-mediated ubiquitination. An increased level of HIF-1α further induces glycolytic genes and enhances hypoxic glycolysis. lincRNA-p21 was recently reported to be significantly elevated in PC cells [218], therefore suggesting a possible link of lincRNA-p21 to hypoxic metabolism in PC.

During rapid, uncontrolled cell division, cancer cell DNA is continuously exposed to various types of damage that lead to genomic instability. Recent studies identified lncRNAs involved in the genotoxic stress response and DNA repair. Prostate-specific lncRNA PCAT1 is upregulated in a cohort of prostate tumor tissues with striking overexpression in a subset of metastatic and high-grade localized tumors [173]. PCAT1 represses the BRCA2 tumor suppressor, causing impaired homologous recombination and DNA repair [203]. This impairment sensitizes cancer cells to PARP1 inhibitors, thus providing PCAT1 the potential to serve as a predictive biomarker of patient responses to PARP inhibitor treatment. Another example of an lncRNA involved in genotoxic stress response is ANRIL, which is also elevated in PC [188]. After DNA damage, ANRIL is induced by the ATM-E2F1 pathway. Expression of ANRIL positively regulates homologous recombination and suppresses expression of the adjacent INK4a, INK4b, and ARF to prepare the cell to re-enter the cell cycle [219]. From a different aspect, TRPM2-AS sustains cell cycle progression in PC by regulating cellular responses to oxidative stress. As an antisense transcript of TRPM2 which encodes an oxidative stress-activated ion channel, TRPM2-AS tightly controls the level of TRPM2 and maintains low intracellular H_2_O_2_. High levels of TRPM2-AS and its related signature genes are associated with poor clinical outcomes, and were suggested to be a potential prognostic marker [216].

### 4.3. lncRNAs in Epigenetic Regulation

Alteration of the epigenetic landscape is one of the key mechanisms allowing PC cells to develop into castration resistance. With epigenetic reprogramming, cells displaying characters such as antiapoptotic and stemness properties may survive after therapy. Remarkably, by far the best-characterized role of lncRNAs is as an epigenetic modulator in gene regulation. As described in the previous section, HOTAIR represses transcription of the AR by forming a complex with polycomb repressive complex 2 (PRC2) on the AR promoter [195]. Similarly, lncRNA-PRC-mediated target repression was also documented for PCAT1 and ANRIL. PCAT1 binds with the PRC2 subunit, suppressor of zeste 12 homolog (SUZ12), and functions predominantly as a transcriptional repressor [173]. ANRIL physically interacts with PRC1 and PRC2 complexes through the subunit CBX7 (chromobox Homolog 7) and SUZ12, respectively, and enhances the recruitment of PRC and methylation of histone 3 lysine 27 on the *INK4b* locus to achieve gene silencing [188,220]. Being overexpressed and associated with aggressive PC, NEAT1 is another example of an lncRNA that modulates gene expression through epigenetic modulation. NEAT1 is upregulated by the estrogen receptor, ERα. Overexpression of NEAT1 alters the chromatin landscape of target promoters with increased levels of H3K4me3 and H3AcK9 via directly binding to the histone, H3, and consequently facilitating active transcription [187]. Given that estrogen signaling through the ERα presents an oncogenic route that bypasses androgen signaling, the ERα-NEAT1-mediated chromatin alteration may drive tumor growth and CRPC progression.

Mechanistically different from the lncRNAs above, SChLAP1 regulates the chromatin status by antagonizing SWI/SNF, a tumor suppressive nucleosome remodeling complex, a homolog of the yeast SWItch/Sucrose Non-Fermentable complex [221]. By interacting with SNF5, SChLAP1 disrupts binding of the SWI/SNF complex to target promoters on a genome-wide scale, resulting in impaired gene expressions [214]. Overexpression of SChLAP1 in PC functionally plays a critical role in promoting invasion and metastasis, and its level independently predicts poor clinical outcomes [214].

### 4.4. lncRNA and miRNA Interplay

In the “competing endogenous RNA” (ceRNA) theory, the seemingly random and chaotic transcriptome is proposed to be a large regulatory network that involves crosstalks between RNAs to achieve gene regulation. In this theory, in addition to the unidirectional targeting of miRNA to mRNA, all transcripts including mRNA, lncRNA and circular RNA (circRNA) that harbor miRNA response elements (MRE) can function as miRNA sponge and compete for binding with the same pools of miRNA, thereby, de-repress the miRNA target genes [222]. The noncoding circRNA CDR1as/CiRS-7 for instance, contains more than 70 binding sites for miR-7 that strongly suppresses the miRNA activity and consequently results in elevation of the target genes [223]. While evidence in PC is scarce, the ceRNA concept has added a new mechanistic dimension of RNA biology to PC research. PTENP1 for example, is a *PTEN* pseudogene whose transcript contains MREs matching the PTEN-targeting miRNAs, miR-17, miR-19, miR-21, miR-26 and miR-124 families [224]. By binding with these miRNAs, PTENP1 increases the cellular level of PTEN and exhibits tumor suppressive activities. Given that PTEN loss is one of the most common alterations in PC that associates with poor clinical outcomes in CRPC patients [225], it is of great interest to understand whether alteration in PTENP1 contributes to downregulation of PTEN in PC, as well as the disease progression. KRASP1, a pseudogene of *KRAS* oncogene, is another example of ceRNA whose expression correlates with the KRAS mRNA level and promotes proliferation in PC cell [224].

## 5. Clinical Implications of ncRNAs in CRPC

Because of the heterogeneous nature of PC, it is challenging to identify patients most likely to benefit from a specific treatment based on PC characteristics at diagnosis. Moreover, there is no curative treatment for PC patients with CRPC and subsequent metastasis, and so innovative treatment options are urgently needed to overcome resistance and improve therapies. Although several prognostic biomarkers for CRPC were reported, due to the drawbacks such as low sensitivity and accuracy and high costs [226], there is still an urgent need to develop and evaluate novel agents to aid therapeutic decisions. Since ncRNA signatures can reflect differences in molecular changes in PC at different stages, levels, and aggressiveness, it appears promising to explore ncRNAs as biomarkers to support the early diagnosis and detection of cases with a poor prognosis, and more importantly, for disease management after the emergence of castration resistance. In addition, ncRNAs play important roles in tumorigenesis, progression, and prognosis of PC, providing a strong rationale for developing ncRNA-based therapeutics by inhibiting overexpressed oncogenic ncRNAs or substituting tumor-suppressive ncRNAs.

### 5.1. Diagnostic Biomarkers

Although the exact cellular source of detectable circulating RNAs is unclear, and some of them may be possibly derived from host defense system in response to therapy or cancer progression, identifying exosomal ncRNAs associated with cancer has become an attractive approach to search for diagnostic and prognostic biomarkers. To date, numerous miRNAs (Table 3) and lncRNAs have been implicated as PC biomarkers that are detectable in circulation, and some of them are correlated with different clinicopathological stages of disease including CRPC. Although many of the proposed markers have not been confirmed or validated in a high-quality manner, the discovery of these circulating ncRNAs has opened an avenue for potential biomarker development and warrants further prospective evaluation.

**Table 3:** Clinical studies investigating the potential of circulating miRNAs as biomarkers of prostate cancer.

Study (Year)	Sample Type and Size	No. of miRNAs Screened	Key Findings
Diagnostic biomarker			
Mitchell *et al.* (2008) [227]	Plasma: 25 metastatic PC patients and 25 healthy controls	6	miR-141 can distinguish patients with PC from healthy controls.
Gonzales *et al.* (2011) [228]	Plasma: 21 PC patients	1	miR-141 was associated with disease progression and changes in PSA.
Zhang *et al.* (2011) [229]	Serum: 50 PC patients (20 localized PC and 30 bone metastasis) and 6 BPH	1	miR-141 was associated with bone-metastatic PC.
Bryant *et al.* (2012) [230]	Plasma: 78 PC patients and 28 normal controls	742	miR-141 and miR-375 were associated with metastatic PC, as well as recurrent disease.
	Serum: 47 recurrent and 72 non-recurrent		
	Urine: 70 local cancer, 48 advanced cancer and 17 normal controls		Urine levels of miR-107 and miR-574-3p were significantly higher in PC compared to controls.
Cheng *et al.* (2013) [231]	Serum: 25 mCRPC patients and 25 healthy controls (cohort 1); 21 mCRPC patients and 20 age-matched healthy controls (cohort 2)	365	miR-141, miR-200a, miR-200c, and miR-375 were higher in mCRPC than all healthy controls.
Kachakova *et al.* (2015) [232]	Plasma: 59 PC patients and two groups of controls: 16 BPH and 11 healthy men	4	let-7c and miR-30c had decreased expression in PC patients compared to BPH patients.
Haldrup *et al.* (2014) [233]	Serum: 31 PC patients (11 localized, 9 metastasis, and 11 CRPC) and 13 BPH controls	732	Identified three miRNA panels for diagnosing and staging of prostate cancer.
Kelly *et al.* (2015) [234]	Whole blood and tissue: 75 PC cancer and 27 BPH	12	A panel of miRNAs (miR-141, miR-145, miR-155, and let-7a) was associated with disease progression and was superior to that of PSA.
Brasa *et al.* (2011) [235]	Serum: 7 metastatic and 14 localized PC patients	668	miRNA-375 and miRNA-141 can identify patients with significantly higher risk of PC.
	Tissue: 36 PC tumors and 36 BPH		
Nguyen *et al.* (2013) [236]	Serum: 28 patients with low-risk localized disease, 30 with high-risk localized disease, and 26 with metastatic CRPC.	667	miR-375, miR-378*, and miR-141 were higher in CRPC compared to low-risk localized patients
Prognostic biomarkers			
Zhang *et al.* (2011)	Serum: 56 patients (20 localized PC, 20 androgen-dependent PC, 10 CRPC receiving docetaxel-based chemotherapy, and 6 BPH)	1	miR-21 was higher in CRPC patients who were resistant to docetaxel chemotherapy.
Li *et al.* (2014) [237]	Serum/plasma: 97 CRPC patients receiving docetaxel chemotherapy	46	Pre-docetaxel miR-200b levels and post-docetaxel changes in miR-20a levels were independent predictors of overall survival of CRPC patients.
Selth *et al.* (2013) [238]	Serum, tissue: 70 BCR and 31 no recurrence PC patients following a radical prostatectomy	4	miR-146b-3p and miR-194 were elevated in RP patients who progressed to BCR.
Sun *et al.* (2015) [239]	Serum: 128 PC patients who received an RP and 100 healthy controls	1	Low miR-128 expression in both PC tissues and patients’ sera had significantly shorter BCR-free survival.

ADPC, androgen-dependent prostate cancer; BCR, biochemical recurrence; BPH, benign prostatic hyperplasia; CRPC, castration-resistant prostate cancer; mCRPC, metastatic CRPC; PSA, prostate-specific antigen; PC, prostate cancer; RP, radical prostatectomy.

#### 5.1.1. miRNA

As potential disease diagnostic biomarkers for PC, individual circulating miR-574-3p, miR-107, and miR-141 were reportedly expressed at higher concentrations in PC patients compared to healthy controls using serum, plasma, and/or urine samples [227,230]. Other than upregulated miRNAs, decreased expression of plasma miR-375 was also shown to be reliable for discriminating PC patients from both BHP and young healthy men with a greater diagnostic performance compared to the current marker, PSA [232]. As single miRNAs might not reflect all characteristics of PC, a panel of four miRNAs consisting of higher levels of miR-141, miR-145, and miR-155 and a lower level of let-7a was shown to have diagnostic potential superior to that of PSA for the detecting PC [234]. In addition, a recent study by Haldrup *et al.* [233] developed three panels of miRNA markers that allow specific identification of PC patients (miR-562/miR-210/miR-501-3p/miR-375/miR-551b), and accurate distinction of patients with disseminated PC from BPH (let-7a*/miR-210/miR-562/miR-616) or organ-confined tumors (miR-375/miR-708/miR-1203/miR-200a). These panels of miRNAs possess the potential to be used for not only diagnosing but also staging of PC. To define CRPC-associated circulating miRNAs, Cheng *et al.* performed miRNA profiling of sera from patients with mCRPC and healthy volunteers across two independent cohorts. Of 365 miRNAs screened, five serum miRNAs (miR-141, miR-200a, miR-200c, miR-210, and miR-375) were detected significantly higher in mCRPC compared to healthy controls [231]. Since miR-141 and miR-375 are the most commonly increased miRNAs in serum of patients with metastatic PC compared to those with localized disease in published studies [230,233,235,236]; therefore these miRNAs might represent useful markers for micro-metastases, with the hope of aiding appropriate selection of patients with organ-confined PC for invasive therapy.

#### 5.1.2. lncRNA

The first lncRNA identified in 1999, DD3 (PCA3), is extremely specific to PC and shows overexpression in >95% of cancer patients [202]. Testing the urinary level of PCA3 showed greater sensitivity, specificity, and malignancy prediction compared to PSA, and thus was developed as a urinary test commercially available to aid diagnoses while avoiding unnecessary biopsies and over-treatment of patients [240,241,242]. While PCA3 urine test has shown promising for the diagnosis of PC patients and prediction of tumor volume, data from two recent independent studies that revealed a gradual reduction and eventual complete non-expression of PCA3 with ongoing hormone treatment [243,244] suggests that urinary PCA3 score are not reliable markers for assessing response to ADT in advanced prostate cancer.

In an RNA-seq analysis of 14 paired-tumor and adjacent benign prostate tissues, metastasis associated with lung adenocarcinoma transcript 1 (MALAT-1) was found to be overexpressed in cancer samples [175], and its expression was correlated with a high Gleason score, tumor stage, and CRPC tumors [198]. Ren *et al.* further showed that a MALAT-1-derived RNA fragment (MD-miniRNA) is a promising plasma-based marker that can improve diagnostic accuracy [245]. Meanwhile, another group reported that MALAT-1 can also serve as a urinary biomarker for high-risk cancer prediction [199].

Recently, the lncRNAs, AK024556 (SPRY4-IT1), FR0348383, and lncRNA-P21, were also reported to be highly expressed in PC and detectable in patient's urine, showing their great potential to be developed as diagnostic biomarkers [218,246,247]. Notably, when compared to parameters including PSA, percent free PSA, and PSA density, FR0348383 appeared to be the most accurate predictor of PC in the subgroup of patients with gray-area PSA (4.0–10.0 ng/mL), and thus, may help avoid unnecessary biopsies while increasing diagnostic sensitivity [214].

### 5.2. Prognostic/Predictive Biomarkers

Relatively less information is available regarding the potential of ncRNAs as prognostic markers for clinical outcomes of PC.

#### 5.2.1. miRNA

In addition to the general overexpression in PC patients, Cheng’s study also showed that serum miR-210 levels are diverse among metastatic CRPC patients undergoing therapy, and it was significantly correlated with the change in PSA level during treatment, an assessment of response to therapy [231]. An elevated concentration of serum miR-194 was reported to possess greater prognostic value of biochemical recurrence (BCR) following a radical prostatectomy [238], and miR-128 was an independent prognostic factor for predicting BCR-free survival [239]. For CRPC patients who received docetaxel chemotherapy, one retrospective study documented that miR-21 was higher in CRPC patients than those with BPH and was associated with docetaxel resistance [106] Another exciting prospective cohort study that recruited 97 CRPC patients receiving docetaxel chemotherapy (75 mg/m^2^ every 3 weeks as one cycle) showed that pre-docetaxel miR-200b levels and post-docetaxel changes in miR-20a levels were independent predictors of overall survival in response to docetaxel chemotherapy [237].

#### 5.2.2. lncRNAs

In a RNA-seq analysis using patient-derived xenografts exhibiting differential metastatic capacities, expression of PCAT18 was found to be incrementally upregulated from normal patients to localized and metastatic cancers. Moreover, given its prostate-tissue specificity and being detectable in plasma, PCAT18 was suggested to be a biomarker for metastatic PC [204]. Similarly, SChLAP1 was significantly overexpressed in metastatic cancers from a cohort of 1008 patient tissues, and was shown to promote tumor cell invasion and metastasis. Although SChLAP1 has not been reported to be detectable in the circulation, its expression was independently associated with 10-year metastasis prediction, and it has a great potential to serve as a tissue-based prognostic biomarker [215,248]. In addition to the two lncRNAs described above, several other lncRNAs, such as DRAIC, GAS5, H19, HOTAIR, Linc00963, PCAT29, and TRPB2-AS that are differentially expressed in CRPC and metastatic cells and are associated with clinical outcomes, may have the potential to be developed as prognostic or predictive biomarkers (Table 2).

### 5.3. Therapeutic Targets

In the field of miRNA research, miRNA mimics are commonly used to restore loss of function, an approach also known as miRNA replacement therapy [249]. Encouraging results from preclinical studies using mouse models demonstrated the promising therapeutic application of miRNA mimics in malignancy revision of PC. For example, systemic delivery of synthetic miR-16 using an atelocollagen-based nonviral vector [250] led to successful downregulation of the therapeutic targets, EZH2 and p110-α, resulting in significant growth retardation of bone metastatic PC3 tumors in mouse bones [251]. Similarly, in two orthotopic PC models with PC3 tumors and LAPC9 tumors, miR-34a complexed with atelocollagen reduced tumor burdens and lung metastases, respectively, which prolonged the overall survival of tumor-bearing mice [126]. Given the broad antioncogenic activity of miR-34a, a liposome-formulated miR-34a mimic (MRX34) is currently in a phase I clinical trial (ClinicalTrials.gov Identifier: NCT01829971) and became the first miRNA-based therapy specifically for cancer [252]. Alternatively, short hairpin (sh)RNAs or pri-miRNA mimics in expression plasmid or viral vectors can be used for therapeutic miRNA replacement. Restoration of let-7c expression by intratumoral injection of let-7c-containing lentiviruses effectively reduced the tumor burden in mice bearing xenografts of human CRPC cells [121]. As discussed earlier, a series of miRNAs such as miR34a, miR-205, miR-488*, and let-7c target the expression of the AR, and some others (Table 1) regulate important cellular metabolism pathways. Turning on expressions of these AR-targeting miRNAs or metabolism-regulating miRNAs could prove to be a useful strategy for combating CRPC by blocking AR-oncogenic pathways or reprogramming metabolism.

On the other hand, miRNA antagonists are designed to block endogenous miRNAs showing a gain-of-function in diseased tissues. As miR-221 is known as a PC-associated oncogenic miRNA, in particular in metastatic CRPC, Mercatelli *et al.* reported the first attempt to use miRNA antagonists to treat PC. In that study, intratumoral delivery of an anti-miR-221/222 antagomiR significantly inhibited the growth of pre-established prostate carcinoma xenografts with a correspondent increase of p27 levels for as long as 25 days after treatment [111]. Although intratumoral administration might not be suitable for clinical use in PC therapy, these results do offer new therapeutic opportunities for evaluation, and set the base for future drug development of antagonists directly against CRPC-associated oncomiRs.

## 6. Conclusions

The era of non-coding RNA research has revolutionized our understanding of cancer biology. Emerging functional roles of aberrant expressions of ncRNAs in human cancers presents unique opportunities to explore their clinical application as a new generation of biomarker for enhancing the diagnosis, prognosis and predicting therapeutic responses of cancer. With advances in genome-wide sequencing technology and updated annotations available for ncRNAs, the number of newly identified ncRNAs associated with PC initiation and CRPC progression is growing fast. On the basis of the encouraging results from preclinical studies, large clinical trials for prospective validation of the many candidate ncRNA biomarkers and their expression signatures as prognostic parameters already identified for PC and CRPC are considered to have high prospects. Comprehensive integration of bioinformatics, clinical informatics systems, and genome and transcriptome databases is expected to strengthen this process.

Traditionally, small-molecule inhibitors were used to target the AR protein with limited success in retarding the progression of PC to metastatic stages. Since CRPC results from activation of multiple pathways, a single treatment strategy would be insufficient to overcome the lethal phenotype of CRPC. Specific ncRNAs have the potential to regulate expressions of several members of a signaling pathway or cellular process, and the coordinated ncRNAs targeting of closely connected genes is prevalent across pathways. Therefore, further knowledge of key regulatory ncRNAs involved in the simultaneous targeting of many pathways, such as the AR signaling axis and cancer metabolic reprogramming, will provide new insights into the development of precise and efficacious anti-CRPC therapies.
